# Supplementary material for: Metagenomic next generation sequencing of plasma RNA for diagnosis of unexplained, acute febrile illness in Uganda
Source: PLoS Negl Trop Dis. 2024 Sep 19;18(9):e0012451. doi: 10.1371/journal.pntd.0012451 (PMC11460704; doi:10.1371/journal.pntd.0012451)
Supplement: Attachment — Submitted filename: Reviewer_PloS_Edited_5232024.docx [file pntd.0012451.s003.docx]

Gabriel Rinaldi, M.D., Ph.D.	23^rd^ May 2024
Academic Editor
PLOS Neglected Tropical Diseases

Elsio Wunder Jr
Section Editor
PLOS Neglected Tropical Diseases

Ref: PNTD-D-24-00106

Dear Dr. Rinaldi and Dr. Wunder Jr,

We thank you and the reviewers for the opportunity to improve manuscript PNTD-D-24-00106. We were able to address all the concerns of the reviewers and revised the text and figures. We have also submitted the metagenomic data to Sequence Research Archive (SRA). For your editorial convenience, we reprinted the reviewer’s comments, then provide our responses and the links to changes in the text/figures.

Please do not hesitate in communicating with us for further ways to improve this work.

Sincerely,

Yukari C. Manabe, MD

Professor of Medicine, International Health, and MMI

**Reviewer's Responses to Questions**

**Reviewer #1**: The study design is clear.  This is more of an exploratory study based on the generation of large genomics data sets rather than hypothesis testing.  The authors aim to evaluate the use of RNA metagenomics in diagnosis for patients with unknown illnesses.  It should be possible to state the study objectives more clearly as a hypothesis, if this is required. Statistical approaches based on Kraken were used, a well-established metagenomics software.

1) It could be further clarified about the sufficient sequencing depth used to perform this study.  Data should be given to summarize the numbers of reads per sample (after mapping to the human genome), and to summarize the z-scores for Pavian in a quantitative manner. Are these z-scores sufficient for the study?  In particular it would be useful to provide the Pavian z-scores for the 9 microbes mentioned in Fig 1 and indicate how they differ from the rest. Were other pathogenic microbes detected by the study, albeit with less significant z-scores?  It would be useful for the authors to provide a full summary of the data, beyond the highlights of interest here, so the reader can get a more general feel for what the method detected, including any background signals.

We thank the reviewer for highlighting the need to clarify our methodology. In the revised manuscript, we now include an additional table (Table 2) listing the read counts per sample after exclusion of human reads. We have also included a supplemental table that lists i) overview of the Pavian results (Suppl Table 1) and ii) KrakenUniq Read classification and KrakenUniq Pavian z-scores (Suppl Table 2).

2) It would probably be helpful to mention the samples were taken from plasma in the abstract.

The revised manuscript now mentions plasma (line 20) in the abstract.

3) The analysis matches the plan. The tables are clear but Figure 1 is of poor quality, and the text is illegible. This needs to be replaced with a higher resolution version.

We apologize for poor quality of figure 1. The revised manuscript includes a better-quality figure 1.

4) The conclusions are supported by the data presented, although some additional detail could be given (see the methods section).
Limitations are clearly described in two paragraphs of the discussion.
The data are clearly connected to public health issues and help with the diagnosis of unexplained illness.
Line 239/240: please clarify DNA pathogens and "transcripts of DNA microbes".  This is currently non-sensical? Is microbial DNA meant?

The reviewer certainly highlights an important observation of our study. In our RNA focused approach, we reduced co-purification of DNA as a contaminant by including a DNase digestion step. Additionally, the Ovation^®^ SoLo^®^ RNA-Seq used in the study included a DNase step to digest any ‘contaminating’ DNA in the RNA input. Hence, the reads that were taxonomically labelled to DNA microbes were likely RNA transcripts. This has previously been observed by others like Kumata et al (PMID:32493363) when they identified transcripts of DNA viruses in the RNA-sequencing dataset from the Genotype-Tissue Expression project. In our study, initial identification of *Rickettsia conorii* transcripts was confirmed using a targeted DNA PCR. We now refer to the study by Kumata et al in the revised manuscript (line 269-270).

5) The authors have applied RNA metagenomics to 42 patients from Uganda with unexplained acute febrile illness. The data were analysed using Kraken, a well-established computational method designed for this kind of study. The data identify pathogenic organisms that had been missed by other approaches, thus demonstrating the utility of the methodology for diagnosis. Some further detail and summary statistics could be given about the analysis, but otherwise this appears to be a fairly routine application of metagenomics to a proof-of-concept study.  It clearly demonstrates the utility of metagenomics for treating patients with unknown illnesses, and has significance for public health issues and medical diagnosis.
The data sets are not yet available, although the authors promise they will be made available on publication.  It is useful for reviewers to check that the correct details and URLs are provided in the manuscript before publication.

The datasets used in this study have been uploaded to Sequence Read Archive (BioProject ID: PRJNA1115716).

**Reviewer #2:** The Objectives of the study are clearly articulated and appropriate. There are no concerns about the ethical conduct of the study per the approvals and adherence standards provided in the Methods.
The study was appropriate for addressing the stated objective to assess the use-case for mNGS as a epidemiologic & diagnostic tool for AFI in settings with diverse potentially diverse and emerging etiologies.
For the question being asked, the sample size is sufficient, and adequate power is not a necessary consideration for this descriptive epidemiologic/diagnostic evaluation.
The use of positive and negative controls, both clinically derived and commercially sourced, is a notable effort for methodologic rigor. The panel-based adjudication process for establishing clinical relevance of an mNGS detection is acceptable approach.

MAJOR
1) Lines 65-67: thrombocytopenia & leukopenia thresholds For the purposes of a reader being able to reconstruct “what was the target patient population?” or “what is the provenance of the participants included in this report?” was there a threshold criterion for thrombocytopenia (e.g., platelets < 100,000?) and was there a threshold criterion for leukopenia (e.g., white blood cell count < 4000?)?

The participants in this mNGS study panel were selected based on i) clinical indication of viral infection (i.e., thrombocytopenia, leukopenia) or sepsis, ii) absence of a validated laboratory diagnosis to explain the clinical presentation, and/or iii) death.

MINOR
2) The study population is appropriate; description of the study population resides in citation 10. To assist the reader’s experience, I recommend the authors include a simple sentence/clause BRIEFLY stating the target population/eligibility criteria: adults with a ≥ 38.0 °C temperature or history of fever within 48 h of presentation (taken from the Abstract of citation 10). Such a sentence will help the reader decide “did the authors aim to recruit a generalizable patient population for this study?” without having to go to PubMed to dig up citation 10.
We agree and have added the following: “In brief, adults (≥18 years of age) in the emergency or outpatient departments to be hospitalized with a recorded fever (≥ 38.0 °C) or clinical history of fever within the past 48 hours who had symptom onset within the past 2 weeks were eligible for enrollment (lines 65-69).

3) Line 78: extraction protocol to reduce DNA contamination.
For the sake of clarity & methods reproducibility, was there a step after centrifugation at 1600g x 15 minutes before filtration using syringe filtration?
In other words, after centrifugation perhaps a certain fraction (the pellet or some other layer) was pipetted and then this fraction was subjected to the additional filtration?

After centrifugation, the supernatant was used for downstream processes. We now include the additional information in the revised manuscript (line 89).

4) Lines 88-90: assessment of library quality prior to sequencing Can the others expand on the criteria used for qualitative assessment of each library by the Bioanalyzer and any criteria used for the quantitative assessment by PCR? For example, was there a minimum quantitative threshold that would have disqualified certain samples from being included on the sequencing run?
We will be happy to explain in more detail. Briefly, the Bioanalyzer was used to determine the average fragment size of the sequencing library and identify presence of non-specific DNA fragments that could potentially affect read quality. For the Ovation^®^ SoLo^®^ RNA-Seq the average library size ranged from 300-350 bp. However, samples that had peaks prior to the library range (likely due to primer dimer or adaptor dimers) were also taken for sequencing as these were smaller (lower concentration) in proportion to the library peak. In our experience these do not affect read quality. Satisfactory libraries were then quantified using PCR and then pooled to a final concentration of 4nM. Required volumes of each sample library was determined using the pooling calculator made available by illumina at https://support.illumina.com/help/pooling-calculator/pooling-calculator.htm. We now include the additional information in the revised manuscript (lines 98-,102,105-107).

5) Lines 115-116: k-mer count.
Can the authors expand on how the “unique k-mer” count from KrakenUniq reports was used?
At present, it is unclear. A reader could speculate that reads mapping to low complexity regions were considered suspicious for spurious detections rather than true infection…?

We thank the reviewer for helping us improve the manuscript. To clarify, reads that hit highly repetitive, low-complexity regions will have very few unique k-mers. e.g., a long (ATATAT...) repeat will only have 2 k-mers. A read of 100bp should have 70 31-mers if they are all different. So, in the KrakenUniq report, if we see, say, 100 reads but only 200 k-mers, that means all of those reads hit the same spot, likely either low complexity or a contaminant. We want the number of uniq k-mers to be much greater than the number of reads. We do not consider a species as a valid finding if the number of reads is not much larger than the number of unique k-mers. This has been included in the revised manuscript (lines 132-136).

6) Lines 116-117: read counts and genome coverage Was there a minimum threshold criterion for number of read counts in order to consider a detection bona fide?
In some publications, I have seen >50 reads used as a threshold. While this might be semi-arbitrary, it would still be helpful to readers in the field to know any thresholds or decision criteria you used in making calls.
From Figure 1 one can see that the number of reads that mapped to R conorii was a small number (and in what turned out to be confirmed by PCR and Sanger sequencing). So if there was not a minimum read threshold, it would still be helpful to state this and/or clarify further the interpretive approach that was used. The same questions apply to genome coverage: was there an operational target that you used?
I realize that with the last 3 comments (library quality assessment, k-mer count, and read count threshold / genome coverage threshold) that there might not be standardized criteria (and perhaps things passed “an eyeball test” (How do I know these were real detections? I know it when I see it.) So these minor queries are not so much meant to be nit-picky as for the sake of Methods reproducibility that would be of interest to the field and to readers who have interest in making platforms like this clinically relevant and actionable.

There was not a minimum threshold for read counts or coverage used to consider microbe detection. We primarily used the z-score calculated to identify potential calls, which were then validated using both the k-mer counts and the coverage by the reads. We did not set a threshold since it can can miss potential hits that yield low but significant reads.

7) HIV Status:HIV infection status is reported in Table 1 of Results, but there was no accompanying explanation in the Methods how this was established: participant self-report? Rapid antibody testing?
Suggest including a sentence towards the beginning of Methods referring the reader to citation 10 for how the demographic/clinical/physiologic information in Table 1 was collected (assuming that citation 10 covers this data collection).

We agree and have added the following: After consent, study personnel collected baseline physiologic parameters, plasma and serum for standardized clinical diagnostic tests including HIV testing with consent (Determine HIV1/2: Abbott, OK, USA; Stat-Pak, Chembio Diagnostics Systems, Inc, NY, USA; SD Bioline: Gyeonggi-do, Republic of Korea), malaria rapid test (CareStart malaria Pf HRP2 antigen RDT), and thick malaria smears as previously described [10]. Participants were followed at one-month through in-person or telephone visits for blood collection and for determining survival (lines 69-74).

8) Figure 2 image file is fuzzy, but hopefully the resolution of a final version will be better.
We apologize for the poor resolution and now include a clearer image.

MINOR
9) Lines 173-175: relevance of P falciparum sequence detections It is arguable whether a detection of P falciparum sequences is indeed clinically relevant in a patient who was smear-negative and malaria RDT negative. The clinical relevance of PCR-based detection of P falciparum is likewise problematic. In other words, this is not a criticism of mNGS, but rather nucleic-acid based detection of P falciparum more generally, as most clinicians would assert that clinically significant P falciparum infection typically includes presence on smear. Was there any evidence that the patient was treated for malaria prior to admission/enrollment?
This detection of P falciparum can still be rendered, in my opinion, as clinically relevant, not so much because malaria would have been the cause of the patient’s illness but because recent or concurrent malaria is associated with non-typhoidal Salmonella (one of the most common causes of meningitis in Africa). I suggest the author’s consider further clarification of this case: whether prior treatment with anti-malarials is known vs unknown and wording that acknowledges it might be problematic to attribute illness to malaria in the context of P falciparum nucleic-acid detection with a negative blood parasite smear.
Molecular detection of *P. falciparum* with a negative blood smear does not implicate malaria as the primary cause of acute febrile illness. However, identification of *P. falciparum* could provide context as an insult that could contribute to anemia, splenomegaly, or non-typhoidal Salmonella infection (lines 252-255). This participant answered ‘unknown’ to the question regarding anti-malarial drugs.

10) Conclusion about MSF in SSA:
The phrasing in the Abstract is technically correct “first report to describe the molecular confirmation of a hospitalized case of MSF in SSA.”
However, in the Conclusion, the rendering is slightly more problematic: “first report of patient admitted with molecularly confirmed locally acquired R conorii infection in SSA.”
AJTMH 2005;73:1086 describes an MSF case acquired in Kenya where the patient returned to Japan and was admitted to hospital in Japan. The Parola et al 2013 global review of rickettsioses (Clin Microbiol Rev 2013;26:657) cites the case of a South African man who acquired MSF in South Africa and then presented with a hemorrhagic fever-like while traveling in Brazil. So I think both of these would be cases of MSF acquired in SSA that required hospital admission.
I recommend the phrasing in the Discussion mirror the phrasing in the Abstract.
Regardless of these semantics, I do think this genetically confirmed detection of MSF in Uganda is significant, as fatal spotted fever rickettsiosis (no speciation) has been described in Kenya and the detection presented in this study is, to my knowledge, only the second genetically confirmed detection of MSF in East Africa, where many assume that the less virulent R africae / African Tick Bite Fever is the main spotted fever rickettsiosis.

We have now edited the manuscript to highlight the significance of *R. conorii* confirmation. Line 233 has been changed to “The findings of this study also include the first report of a molecularly confirmed locally acquired *R. conorii* infection leading to hospitalization in SSA.”

11): Overall, I think this work adds value to the field 1) showing the strengths (unbiased nature) and limitations (diminished sensitivity and overall low detection frequency) of mNGS as a surveillance tool. 2) The detection of R conorii is a public health relevance as this is a neglected disease and severe disease is more common with R conorii than with R africae.
As stated in my comments above, I think the manuscript would benefit from some additional linkage to the parent study (e.g., providing the inclusion criteria), expanding on some methodologic details (namely for mNGS interpretive methods but also providing details of the selection criteria for thrombocytopenia and leukopenia). 3) I have suggested some nuance around the novelty of the R conorii detection in Uganda.
We thank the reviewer for their helpful and encouraging comments. As suggested, the revised manuscript now contains more information on the parent study (lines 65-74), methodological details (lines 89,98-107,132-138), and novelty on the *R.conorii* detection(line 233).
