# Supplementary material for: Metagenomic next generation sequencing of plasma RNA for diagnosis of unexplained, acute febrile illness in Uganda
Source: PLoS Negl Trop Dis. 2024 Sep 19;18(9):e0012451. doi: 10.1371/journal.pntd.0012451 (PMC11460704; doi:10.1371/journal.pntd.0012451)
Supplement: Attachment — Submitted filename: Reviewer_PloS_Edited_07232024.docx [file pntd.0012451.s004.docx]

Gabriel Rinaldi, M.D., Ph.D.	23^rd^ July 2024
Academic Editor
PLOS Neglected Tropical Diseases

Elsio Wunder Jr
Section Editor
PLOS Neglected Tropical Diseases

Ref: PNTD-D-24-00106R1

Dear Dr. Rinaldi and Dr. Wunder Jr,

We thank you and the reviewers for the opportunity to further improve manuscript PNTD-D-24-00106R1. We appreciate the reviewers’ comments and have responded to the remaining queries. For your editorial convenience, we copy pasted the reviewers’ comments, then provide our responses and the links to changes in the text/figures.

Please do not hesitate to contact us if you have additional questions.

Sincerely,

Yukari C. Manabe, MD

Professor of Medicine, International Health, and MMI

**Reviewer's Responses to Questions**

**Reviewer #1**:
The authors have left the phrase "DNA microbes" in the paper.  I find this phrase confusing.  It is clear from their comment that they mean DNA viruses - which is perfectly sensible.  But do they intend to include other microbes?  It surely makes no sense to talk about DNA bacteria for instance.  Would it be clearer to change "DNA microbes" to "DNA viruses"?  The paper they now cite from Kumata et al only appears to refer to DNA viruses, I could not find "DNA microbes" in that paper. However, I will leave this issue to the authors discretion.

We apologize for the confusion. To clarify, although we enriched for virions during RNA extraction, the protocol allowed us to simultaneously sequence circulating cell free RNA transcripts of other DNA microbes like viruses, bacteria, fungi, and protozoa. We have now clarified this in the text (lines 286-287).

The authors state that the data have been uploaded under project ID PRJNA1115716, but this ID gives no hits on the SRA or NCBI.  Perhaps it is incorrect or not yet released?  The PLOS Data policy requires authors to make all data underlying the findings described in their manuscript fully available without restriction, and so I assume the paper should not be published until this has been sorted out.

As per PLOS Data policy the data have been deposited. The data will be released on 22^nd^ Nov 2024 or with the release of the linked data, whichever is first.

**Reviewer #2:**

The authors have responded adequately to my concerns and suggestions. Of note, the line numbering in their responses did not align with the revised manuscript version I received, so it did make the process of reviewing their responses more onerous than one would have hoped. Overall, though, their additions and edits have improved the manuscript and increased its informativeness.

We are glad to know that we have responded to your concerns and suggestion. We apologize for the confusion over the line numbers.
